# Variation in local population size predicts social network structure in wild songbirds

**DOI:** 10.1111/1365-2656.14015

**Published:** 2023-10-13

**Authors:** Kristina B. Beck, Damien R. Farine, Josh A. Firth, Ben C. Sheldon

**Affiliations:** ^1^ Department of Biology, Edward Grey Institute University of Oxford Oxford UK; ^2^ Department of Evolutionary Biology and Environmental Studies University of Zurich Zurich Switzerland; ^3^ Division of Ecology and Evolution, Research School of Biology Australian National University Canberra Australian Capital Territory Australia; ^4^ Department of Collective Behaviour Max Planck Institute of Animal Behaviour Konstanz Germany

**Keywords:** great tit, habitat configuration, population size, simulation, social network, social structure, vegetation

## Abstract

1. The structure of animal societies is a key determinant of many ecological and evolutionary processes. Yet, we know relatively little about the factors and mechanisms that underpin detailed social structure.
2. Among other factors, social structure can be influenced by habitat configuration. By shaping animal movement decisions, heterogeneity in habitat features, such as vegetation and the availability of resources, can influence the spatiotemporal distribution of individuals and subsequently key socioecological properties such as the local population size and density. Differences in local population size and density can impact opportunities for social associations and may thus drive substantial variation in local social structure.
3. Here, we investigated spatiotemporal variation in population size at 65 distinct locations in a small songbird, the great tit (*Parus major*) and its effect on social network structure. We first explored the within‐location consistency of population size from weekly samples and whether the observed variation in local population size was predicted by the underlying habitat configuration. Next, we created social networks from the birds' foraging associations at each location for each week and examined if local population size affected social structure.
4. We show that population size is highly repeatable within locations across weeks and years and that some of the observed variation in local population size was predicted by the underlying habitat, with locations closer to the forest edge having on average larger population sizes. Furthermore, we show that local population size affected social structure inferred by four global network metrics. Using simple simulations, we then reveal that much of the observed social structure is shaped by social processes. Across different population sizes, the birds' social structure was largely explained by their preference to forage in flocks. In addition, over and above effects of social foraging, social preferences between birds (i.e. social relationships) shaped certain network features such as the extent of realized social connections.
5. Our findings thus suggest that individual social decisions substantially contribute to shaping certain social network features over and above effects of population size alone.

## Graphical abstract

The authors show how habitat features shaped variation in local population size in great tits, and how population size subsequently affected social structure. However, much of the observed social structure was still shaped by social processes such as the birds' preference to forage in flocks and social relationships between them.

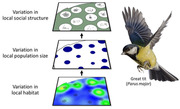

## 1 INTRODUCTION

Animal social structure emerges from the patterns of social associations and interactions between individuals (Hinde, 1976; Wilson, 2000). Social structure can take many different forms (Prox & Farine, 2020; Sah et al., 2018) and fundamentally impacts individual fitness in many ways, for example by shaping the acquisition of (extra‐pair) mating partners (Beck et al., 2021; Oh & Badyaev, 2010; Wey & Blumstein, 2012) and survival (Alberts, 2019; Silk et al., 2003; Stanton & Mann, 2012), as well as influencing several population processes such as the transmission of diseases and information (Aplin et al., 2012; Evans et al., 2020; Sah et al., 2017), and the direction and intensity of selection (McDonald & Pizzari, 2018; Montiglio et al., 2018). Thus, gaining a deeper understanding of the factors and mechanisms underlying the variation in animal social structures is important for comprehensively elucidating the implications and evolutionary trajectories of sociality in animals (Cantor et al., 2020).

An individual's social connections are a crucial component of its environment, which is often referred to as its ‘social environment’. The formation and maintenance of social associations and relationships can be challenging, requiring careful investment of time and energy. Social structure arises as a consequence of how individuals navigate these challenges and their decisions about whom to interact with (Cantor & Farine, 2018; Hinde, 1976; Strandburg‐Peshkin et al., 2017) and the opportunities that individuals have to establish social bonds (Goldenberg et al., 2016; Ilany & Akcay, 2016; Ilany et al., 2021). For instance, in vampire bats ( *Desmodus rotundus* ), the formation of high‐cost cooperative bonds between non‐kin is mediated by prior low‐cost grooming interactions, demonstrating how investment (e.g. grooming) is required for the establishment and maintenance of social bonds (Carter et al., 2020). Individuals might also have preferences for associating with particular conspecifics, for avoiding certain conspecifics, or for associating with particular types of conspecifics (e.g. assortativity or disassortativity). For example, female Masai giraffes (*Giraffa camelopardalis tippelskirchi*) live in fission–fusion societies but often preferably associate with kin (Carter et al., 2013), and in bottlenose dolphins (*Tursiops aduncus*) community membership is correlated with the dolphins' response to fishing activity, whereby individuals that feed in association with humans are also more likely to be part of the same community (Louise Chilvers & Corkeron, 2001).

While social decisions by individuals shape who they have contact with and form social relationships with, it is less clear how social structure at a larger—for example, population scale—emerges, and whether differences among groups or populations are the result of purely individual‐level decisions or broader ecological factors. Evidence from semi‐wild replicated stable social groups suggests that different sets of individuals might express consistent differences in overall social structure (Van Leeuwen et al., 2018). Social structure could also be influenced by features of the physical environment in which social associations take place. Specifically, habitat configuration, such as physical barriers (e.g. mountains, rivers, streets) and the availability and distribution of food resources, plays an important role in shaping not only the number of animals, but also when and where animals can move to and from, therefore, who can socialize with whom (He et al., 2019, 2021; Lantz & Karubian, 2017; Leu et al., 2016; Webber et al., 2023). When habitat components (e.g. habitat patches) are heterogenous, such as when they vary in resource quality or quantity across different spatiotemporal scales, they might also shape population demographic factors that contribute to social structure (He et al., 2021; Tokeshi & Arakaki, 2012). For instance, areas with more resources could support a larger number of individuals (i.e. larger population size), or those with greater temporal variability could underpin larger population turnover (Brown & Orians, 1970; Emlen & Oring, 1977). Thus, heterogeneity in a landscape could fundamentally impact opportunities for social encounters between individuals and subsequent local social structure.

Population density, that is the number of individuals per spatial unit, is largely driven by resource distribution, and is known to mediate many social processes in animal populations, such as reproduction (Kokko & Rankin, 2006; Leary et al., 2008; Niemelä et al., 2021) and the transmission of diseases (Albery, 2022; Anderson, 1982; Hopkins et al., 2020). For example, diseases are commonly expected to spread more rapidly when population density is high because individuals should be more likely to encounter an infected conspecific (Mariën et al., 2020; Storm et al., 2013). By shaping not just which, but also how many, conspecifics can be encountered, population density (or population size) should also have an impact on social network structure. However, despite the wealth of studies on density dependence that link population density or social measures such as group size to the population process of interest (e.g. disease transmission), we still know surprisingly little about how variation in population density actually shapes the social connectivity among individuals and the consequences that this has on the animal social networks themselves.

Population density should naturally translate to differences in key parameters of social structure by influencing opportunities for social associations. For example, as with the ideal gas law, where the collision rate of molecules increases with the concentration of molecules in the gas (Maxwell, 1860), increases in the number of individuals in a given area is expected to increase the density of social contacts (Hutchinson & Waser, 2007; Webber et al., 2023). In a social context, this can translate to larger group sizes (Caughley, 1964; Pépin & Gerard, 2008; Vander Wal et al., 2013; Webber & Vander Wal, 2021), with correspondingly many effects on other social network properties, such as measures of centrality (Newman, 2018). A range of studies demonstrate how variation in group size can shape social structure (Balasubramaniam et al., 2014; Maldonado‐Chaparro et al., 2015; Nunn et al., 2015; Webber & Vander Wal, 2020). For instance, in yellow‐bellied marmots (*Marmota flaviventris*), network density decreased with increasing group size (Maldonado‐Chaparro et al., 2015), as individuals may have a limited capacity to establish more social connections as the availability of conspecifics increases (e.g. due to time constraints, García et al., 2021 or cognitive constraints, Dunbar, 1992). Interspecies comparisons have also suggested that network fragmentation (modularity) generally increases with increasing group size (Nunn et al., 2015). However, individuals rarely move like molecules in a gas and increases in population size and density may thus not necessarily impact the extent of social contacts (Hutchinson & Waser, 2007). For instance, animals are often territorial and actively avoid others, they preferably forage with (certain) conspecifics (e.g. social foraging), and they exhibit different degrees of sociality and group cohesion (from relatively solitary, to gregarious, and socially stable groups, Sah et al., 2018). This raises the question to which extent social decisions (such as the preference to socially forage) and simply adding more individuals in space (population size and density) shape the observed social structures.

Here, we first investigated the effect of habitat characteristics on the spatiotemporal variation in local population size in a small songbird, the great tit (*Parus major*). Subsequently, we examined the consequences of the observed variation in population size on the birds' social network structure. During the non‐breeding season in winter, great tits forage in loose fission–fusion flocks (Perrins, 1979). We analysed a unique dataset with information on habitat features, and the birds' social associations and local population size at 65 distinct locations across multiple weeks spanning 3 years. These data allowed us to examine differences in local population size on a relatively small spatial (at each location) and temporal scale (in each week). We first examined the extent to which local population size, defined here as the number of unique great tits visiting a feeder location, is consistent at each location across time. We then asked whether underlying habitat features at the feeder location, that is the shrub‐cover density, explain variation in local population size. We predicted that locations with denser shrub cover will support higher numbers of birds. This is because denser shrub cover should offer better protection against predators while foraging (Dagan & Izhaki, 2019; Díaz, 2006; Quinn et al., 2012).

We then investigated whether differences in local population size shape local social structure in foraging great tits using social network analysis. We focused on the relationship between population size and four global social network metrics depicting different features of the weekly sampled social network structure at each location: network edge density, global clustering coefficient, mean edge weight and modularity (for details, see Methods—social network structure). They measure respectively the extent to which individuals are connected to others, the extent to which individuals tend to cluster together, the average strength of connections to others and the extent to which the network is fragmented into subgroups. Finally, we asked how the relationship between population size and each network metric deviated from random expectations by performing two empirically driven models that eliminated different social features (Farine et al., 2015). First, we ran a ‘simple’ model that simulated foraging visits to a single location of varying numbers of individuals (i.e. varying population size), ignoring the birds' preference to socially forage. Second, we performed a ‘social foraging’ model where we kept the observed flock sizes at a distinct location constant (thus maintaining the birds' preference to forage in flocks) but swapped individuals between flocks in order to disrupt their social preferences with whom to forage with. Therefore, these swaps altered the observed social network structure by changing who was connected to whom and how strong (i.e. the birds' social relationships). We then compared the relationship between local population size and the global network metrics of the two models to the observed relationship. This allowed us to examine to what extent population size alone explained differences in social network structure (simple model), and to what extend social features such as the birds' preference to forage in flocks (social foraging model) and social relationships (observed data) shaped the social network structure. As such, we aim to highlight the importance of habitat configuration and spatiotemporal variation in local population in governing social structure in natural settings.

## 2 MATERIALS AND METHODS

### 2.1 Study species and system

Great tits are short‐lived, hole‐nesting songbirds that form socially monogamous pairs (Perrins, 1979). Great tit pairs establish territories during the breeding season (April–June) and form loose fission–fusion flocks of variable size and composition during autumn and winter that often consist of mixed species (Perrins, 1979).

We studied a population of great tits located in Wytham Woods, Oxfordshire, UK (51° 46′ N, 01° 20′ W). The study site spans about 385 ha and consists of largely broadleaf deciduous woodland surrounded by open farmland (Savill et al., 2011). Great tits were caught in either a nest box or a mist‐net and were fitted with a uniquely numbered metal ring (British Trust for Ornithology, BTO). Furthermore, over 90% of tits in the study site, were also fitted with a uniquely coded passive integrated transponder (PIT) tag enclosed in a plastic ring (Aplin et al., 2013). During the winter month over 3 years (December 2011–March 2014), 65 bird feeders were deployed in an evenly spaced grid (Figure S1). Each feeder contained two access holes equipped with radio‐frequency identification (RFID) antennas and was placed inside a meshed metal cage, large enough to allow great tits to access the feeder but small enough to prevent larger animals from exploiting food and damaging the equipment. Whenever a PIT‐tagged bird landed on the RFID antenna of a feeder, its unique PIT tag code, and the date and time were saved to a data logger. The feeders collected data from pre‐dawn Saturday morning until after dusk on Sunday evening resulting in 39 weekly samples (13 weekends each year). Data were only collected for 2 days per week to capture a synchronous snapshot of social structure while minimizing the potential effect of feeding on population dynamics (e.g. survival rates, movements) and social structure. Weekends were chosen for logistical reasons (e.g. to perform feeder maintenance during weekdays). This dataset on the foraging associations of birds, forms part of a study investigating the social ecology of great tits and provided numerous insights into, for example, individual sociality (Aplin et al., 2015), social structure (Farine et al., 2015) and its' links to ecological processes such as information transmission (Aplin et al., 2012; Firth et al., 2016), breeding settlement (Firth & Sheldon, 2016) and mating behaviour (Culina et al., 2015).

All work was subject to review by the University of Oxford, Department of Zoology, Animal Welfare and Ethical Review Board (Approval number: APA/1/5/ZOO/NASPA/Sheldon/TitBreedingEcology). Data collection adhered to local guidelines for the use of animals in research and all birds were caught, tagged and ringed by appropriate BTO licence holders.

### 2.2 Repeatability of local population size

**FIGURE 1:**
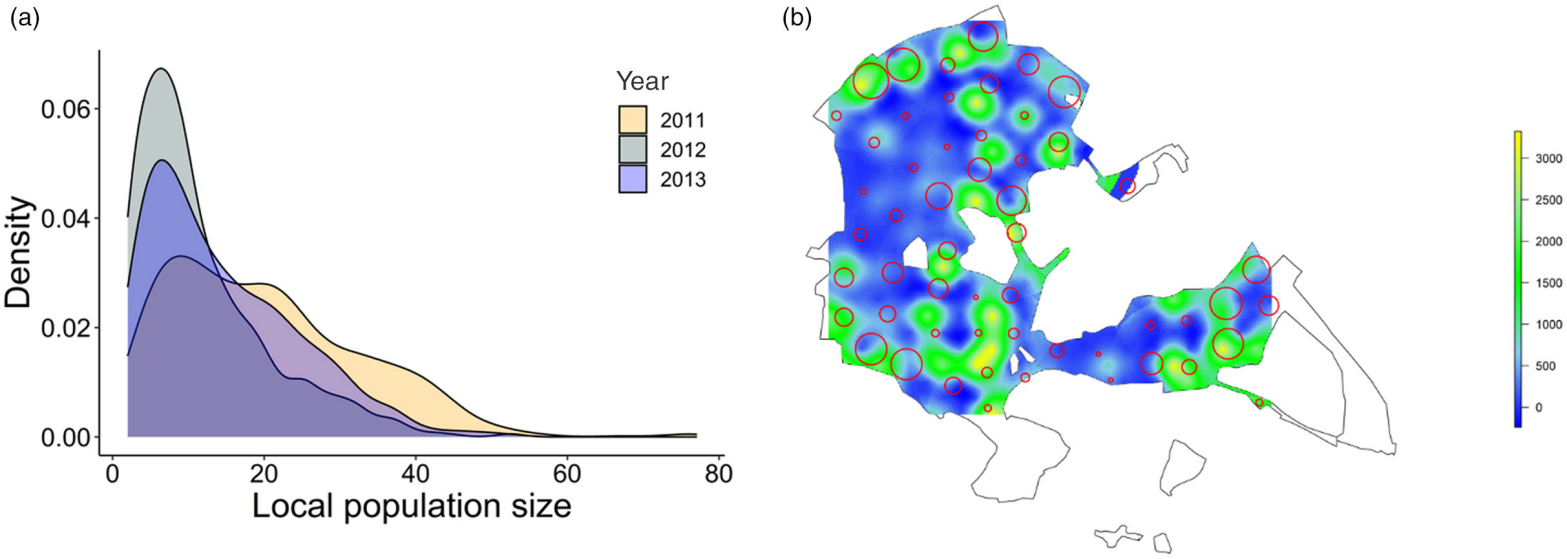
(a) Data distribution of weekly, local population size for each year separately (2011, 2012, 2013). (b) Map of the study site with red circles indicating the locations of feeders included into the analysis on shrub‐cover density. Circle size indicates the average population size for each feeder location (weekly averages across the 3 years). The coloured background shows the extrapolated shrub‐cover density (yellow: high density, blue: low density).

All data manipulation and statistical analysis were performed in R studio version 4.3.0 (R Core Team, 2020). Data on the weekly bird visits to the different feeder locations show considerable variation in local population size (Figure 1). We define local population size as the number of unique individual great tits recorded at a given location (i.e. feeder) and week. Repeatability was computed as the proportion of the variance explained by the ‘location' within a GLMM using the ‘rptR’ package (Schielzeth & Nakagawa, 2013). Local population size was set as the response variable (Poisson distribution), with one measurement per location and week. We set the location identity (i.e. feeder identity) as a random effect and included week (continuous variable ranging from 1 to 13 reflecting the week since beginning of data collection in each year) and year (categorical variable for winters 2011, 2012 and 2013) as fixed effects to account for temporal trends within and across years.

Statistical significance of the repeatability estimate was inferred using permutation tests. Specifically, we carried out 1000 permutations of the weekly datasets, whereby we shuffled each of the location identities randomly within each year and week. This kept the distribution of observed local population sizes during a given year and week constant but reallocated the observed sizes to random locations, thereby breaking the link between population size and feeder location. We then re‐calculated the repeatability estimate using the same model (but on the randomized dataset), and then assessed the significance of the observed repeatability as a one‐tailed *p*‐value from its place in the null distribution (randomly generated repeatability coefficients), where *p* < 0.05 would indicate that the observed repeatability statistic was larger than expected by chance (i.e. larger than the 95% range of the null distribution of the repeatability statistic).

### 2.3 Habitat features and local population size

We used data from Kirby et al. (2014) to quantify the vegetation structure at each feeder location. From 1974 to 2012, the authors recorded shrub‐cover density (all vegetation 0.5–2.5 m above‐ground) along the diagonal of 164 different 10 m × 10 m quadrats across Wytham Woods (Figure S1; Kirby et al., 2014). In our study, we used data from the 2012 census since this period most likely represents the shrub‐cover density during our study period. To infer shrub‐cover density from the entire study site, we generated a surface plot and extrapolated from the 164 sites to a 10 m × 10 m grid of points spanning the whole study site using the package ‘gstat’ (Pebesma et al., 2015) with a spherical model, omitting fitting the nugget component (following the approach in Farine & Sheldon, 2019). We then created a 30 m radius around each feeder location and extracted the mean estimated shrub‐layer density from the extrapolated surface (i.e. the 10 m × 10 m grid) within that radius using the package ‘raster’ (Hijmans, 2023). On average 28 extrapolated measures for shrub‐layer density (i.e. 28 grid cells) contributed to the inferred mean shrub‐layer density for each feeder location. We repeated the analysis with a 100 m radius.

We fitted a GLMM using the ‘lme4’ package (Bates et al., 2015) with the weekly, local population size at a feeder location (i.e. weekly number of birds recorded at a location, with a truncated negative binomial error distribution) as the response variable. We fitted shrub‐layer density as fixed effects. In addition, we fitted the distance to the edge of the study site as fixed effect because a previous study reported a positive relationship between population size and proximity to the forest edge (Wilkin et al., 2006). Location identity and week nested within year were fitted as random effects. The fixed effects shrub‐layer density and distance to the forest edge were standardized by subtracting the mean and dividing the standard deviation. Both fixed effects did not correlate substantially (*r* = −0.24) and we validated model fit by visually investigating residual and qq plots. We tested for temporal and spatial autocorrelation using the package ‘DHaRMa’ (Hartig, 2021) which suggested no contributing issues in our models in this context.

### 2.4 Social structure

#### 2.4.1 Social network construction

Following previous work on this system, for each sampling week and feeder location, we created social networks based on the foraging associations of PIT‐tagged great tits. Because birds forage in flocks, the records of visits will typically consist of periods with high activity (when birds forage at the feeder) and periods of no activity (when birds are absent) (see fig. 2 in Psorakis et al., 2012). We used the package ‘asnipe’ (Farine, 2013) to assign individuals to flocking events using Gaussian mixture models (Psorakis et al., 2012, 2015). The package detects periods of increased feeding activity in the data and clusters these periods into non‐overlapping gathering events (i.e. flocking events), without using arbitrary temporal boundaries defining flock membership. We recorded on average 87 flocking events for a given location and week, lasting on average 4 min. Individual visits were then assigned to the corresponding flocking event creating a group (i.e. flock) by individual matrix. This matrix includes information on each flocking event (rows) and which individuals (columns) were present in each flock. Following, we defined an ‘association’ as two birds co‐occurring in the same flock and inferred the association strength between individuals as the proportion of observations containing at least one individual also contained the other (the simple ratio index or SRI, Cairns & Schwager, 1987). The SRI ranges from 0 (never observed together) to 1 (always observed together). We created undirected social networks (because associations between individuals were symmetrical), with edges (i.e. connections between individuals) weighted by the SRI for each location (*N* = 65) and weekly sample (*N* = 39 across 3 years).

#### 2.4.2 Social network structure

We calculated four commonly used global network metrics using the package ‘igraph’ (Csardi & Nepusz, 2006) to characterize the structure of each weekly, local network: network edge density, global clustering coefficient, modularity and the average edge strength. We selected these metrics with the aim to describe different dimensions of the social network. The (binary) network edge density was calculated as the number of existing connections divided by all potential connections and gives a measure for how well individuals of a network are connected overall. Higher values of network edge density indicate that individuals in a network have a greater tendency to be connected to others in the network. The global clustering coefficient was defined as the ratio of the triangles and the connected triples within the network and measures the extent to which individuals tend to cluster together. It thus measures connectivity among individuals at a more local level than for instance network edge density or modularity. A high global clustering coefficient indicates that individuals tend to be more clustered together. Modularity describes the separation of networks into structural subgroups of individuals that have more connections among themselves than they do with individuals outside of that subgroup. We inferred the structural communities (modularity index *Q*) for each network using the edge betweenness community detection algorithm. Values of 0 indicate that connections are random and values below 0 suggest that nodes do not form clear communities. Positive values of modularity index *Q* indicate that a subgroup has more connections than expected by chance. Thus, larger values suggest a larger separation into subgroups or communities. For example, if we imagine two networks consisting of 20 individuals each, but one with a modularity value of 0.05 and one with 0.2, the latter would be separated into more distinct communities (e.g. eight compared to five communities). Finally, we calculated the average edge strength defined as the average of all non‐zero edge weights in a network and provides a measure for how strong the existing connections between individuals in a network are. Higher values of weighted average strength indicate that individuals have stronger connections among each other.

### 2.5 Local population size and local social structure

#### 2.5.1 Observed relationship between population size and social structure

We examined the effect of local population size on measures of local social structure by fitting (G)LMMs using the package ‘lme4’ (Bates et al., 2015). In our dataset, several social networks were fully connected, that is every individual was connected to everyone else (i.e. network edge density of 1, Figure S2). Therefore, we first investigated how overall ‘network connectivity’ (in terms of whether they are fully connected or not fully connected) was predicted by local population size (*N*_Networks_ = 2232). We performed a binomial GLMM with network connectivity (‘network is fully connected’ vs. ‘network is not fully connected’) as the response variable. As fixed effects, we fitted the log‐transformed local population size, and the location identity and week nested within year as random effects. In a next step, we excluded all fully connected networks (remaining *N* = 1992) and examined the effect of population size on network edge density. We fitted the square‐transformed edge density as the response variable in a linear‐mixed effect model (LMM) with the same fixed and random effects as described above. For the analysis on global clustering coefficient, mean edge weight and modularity, we removed cases where less than two connections between individuals (two edges) were present (remaining *N* = 1939). Following, we fitted separate LMMs with the square‐transformed global clustering coefficient, and log‐transformed mean edge weight as response variables. For modularity, the distribution of values was inflated by values close to 0 (i.e. values > −6.66e‐15 and <=0.0001, Figure S3). Therefore, we first performed a binomial GLMM aiming to examine the relationship between ‘zero’/’non‐zero’ modularity and local population size. We modelled values of modularity close to 0 (i.e. all values <=0.0001) versus modularity values larger than 0.0001 as the response variable. Next, we excluded modularity values close to 0 (remaining *N* = 1329) and fitted the log‐transformed modularity as the response variable in a LMM. Fixed and random effects were the same as described above. We validated model fit by visually investigating residual and qq plots, and tested for temporal and spatial autocorrelation using the package ‘DHaRMa’ (Hartig, 2021).

The method to infer flocking events (i.e. Gaussian mixture models Psorakis et al., 2012; Psorakis et al., 2015) and subsequently calculate social associations sometimes erroneously clusters individual visits over a long period of time into the same event. In such cases, the duration for a flocking event might be estimated to last several hours (max = 9.3 h). The average duration of flocking events is estimated to last 4 min and overestimations of, for example, several hours are rare (the 99% quantile of all estimated flocking durations corresponds to 12 min). However, to avoid biases by unnaturally long estimated flocking events in our calculated social networks, we excluded networks including flocking events lasting longer than 12 min and repeated the analysis as described above.

#### 2.5.2 Comparing the observed to theoretical relationships between population size and social structure

To investigate how population size alone is expected to shape social structure, we performed a ‘simple’ model ignoring any social factors (e.g. the birds' preference to forage in flocks and their preference to forage with specific individuals; Psorakis et al., 2012). We modelled different population sizes by randomly selecting *N* individuals' data from random locations and random weeks. *N* reflected the weekly, local observed numbers of visiting great tits (i.e. local, weekly population size). This kept the time of day that individuals would forage constant but removed any social factors. We then created social networks from these randomly generated foraging associations in the same way as for the observed foraging associations (see Section 2.4.1). We repeated this process for each population size ‘N’, 100 times. The ‘simple’ model assumes no social attraction between birds. However, great tits prefer to socially forage in flocks (Psorakis et al., 2012). Therefore, we also performed a ‘social foraging’ model where we maintained the observed flock sizes and the temporal distribution of visits during the day at a given location but disrupted social preferences between birds. To do so, we performed a permutation on the group by individual matrix (see Section 2.4.1) by randomly swapping individuals between flocks recorded at the same location and week. This disrupted the social relationships between dyads but maintained the flock sizes and the temporal visitation patterns observed within each week and location. We repeated this process 100 times. This allowed us to explore to which extend social foraging alone (without any social preferences with whom to associate) shape global social network structure. Finally, we compared the relationship between local population size and social network metrics between the observed data, and the ‘simple’ and ‘social foraging’ model.

## 3 RESULTS

### 3.1 Repeatability of local population size

We recorded a total of 1823 individual great tits across the 3 years, with the majority of birds foraging at only one feeder location during a given sampled week (min = 1, mean = 1.48, max = 11). We inferred 2232 weekly, local measures of population size. Weekly, local population sizes ranged from 2 to 77 individuals (mean = 15.35, Figure 1a) and were highly repeatable within locations across weeks and years (repeatability coefficient *R* = 0.66). The repeatability coefficients generated from 1000 permutations of the weekly datasets, whereby we shuffled each of the location identities randomly within each year and week were all close to 0 (range: 0–0.01), capturing how much greater the observed repeatability was than expected by chance (*p*‐value < 0.001).

### 3.2 Habitat features and local population size

We examined whether features of the habitat, that is the underlying shrub‐layer density and the distance to the forest edge, predicted local differences in population size (Figure 1b). For this analysis, we only included those feeder locations for which shrub‐cover data from the 2012 survey by Kirby et al. (2014) were available (*N* = 54; Figure 1b, Figure S2). We found no evidence that shrub‐layer density predicted local population size (estimate ± SE: 0.06 ± 0.08; Table S1, Figure S4). However, feeder locations closer to the forest edge had on average higher population sizes (−0.19 ± 0.08; Table S1, Figure S4). Repeating the analysis with a 100 m radius did not substantially change the results (Table S2).

### 3.3 Local population size and local social structure

#### 3.3.1 Observed relationship between population size and social structure

We examined whether variation in local population size affected the local great tit social network structure. We first investigated the effect of local population size on overall network connectivity, that is differentiating between networks that were fully connected and those that were not. We found that fully connected networks were less likely to occur at locations with larger population sizes (estimate ± SE: −2.90 ± 0.21; Table 1, Figure 3a) and the majority (95%) of fully connected networks coincided with local population sizes of 14 or fewer individuals (mean = 4.8 individuals). Network edge density and global clustering coefficient increased with increasing population size (network edge density: 0.08 ± 0.01; global clustering coefficient: 0.14 ± 0.01; Table 1, Figure 3b,c) while mean edge weight decreased with increasing population size (−0.17 ± 0.01; Table 1, Figure 3d). Modularity was generally close to zero (Figure S3). Therefore, we first examined whether population size affected the probability of modularity being close to zero versus larger (i.e. <=0.0001/>0.0001). Modularity close to zero was much more likely at locations with small population sizes (2.50 ± 0.15; Table 1, Figure 3e) with 95% of networks with modularity close to zero being at locations with 21 or fewer individuals. When modelling non‐zero modularity values (i.e. >0.0001), we found that modularity decreased with increasing population size (−0.94 ± 0.08; Table 1, Figure 3f), suggesting that social networks are more fragmented. Network edge density and global clustering coefficient were positively correlated and network edge density and modularity negatively correlated (Table S3). Excluding networks containing flocking event durations longer than 12 min did not substantially change the results (Table S4).

**TABLE 1:** Results of the (G)LMMs examining the effect of local population size on the observed social network structure (network connectivity, square‐transformed network edge density, square‐transformed global clustering coefficient, log‐transformed mean edge weight, modularity (binomial, (<=0/>0)) and log‐transformed modularity (for values >0)). Shown are estimates ± standard errors (SEs), the test statistic (i.e. *z* and *t* statistics), 2.5% and 97.5% confidence Intervals (CIs) and the *p*‐value (*p*). Location and week nested within year were set as random effects (variance and standard deviation: Model on network connectivity: Location = 0.23, 0.48; week:year = 0.20, 0.44; model on network edge density: Location = 0.01, 0.10; week:year = 0.002, 0.04; model on clustering coefficient: Location = 0.004, 0.07; week:year = 0.001, 0.03; model on mean edge weight: Location = 0.01, 0.10; week:year = 0.01, 0.10; model on modularity (<=0/>0): Location = 0.30, 0.55; week:year = 0.00, 0.00; model on modularity (>0): Location = 0.28, 0.53; week:year = 0.03, 0.18).

	Estimate ± SE	Test statistic	2.5% CI	97.5% CI	*p*
Network connectivity					
Intercept	3.16 ± 0.48	6.64	2.22	4.09	<0.001
Population size^a^	−2.90 ± 0.21	−13.86	−3.31	−2.49	<0.001
Network edge density					
Intercept	0.29 ± 0.05	6.16	0.19	0.39	0.003
Population size^a^	0.08 ± 0.01	8.53	0.06	0.10	<0.001
Clustering coefficient					
Intercept	0.31 ± 0.04	8.10	0.23	0.38	<0.001
Population size^a^	0.14 ± 0.01	15.36	0.12	0.16	<0.001
Mean edge weight					
Intercept	−1.40 ± 0.05	−29.93	−1.49	−1.31	<0.001
Population size^a^	−0.17 ± 0.01	−13.51	−0.20	−0.15	<0.001
Modularity (<=0/>0)					
Intercept	−5.36 ± 0.38	−14.06	−6.11	−4.62	<0.001
Population size^a^	2.50 ± 0.15	16.71	2.21	2.80	<0.001
Modularity (>0)					
Intercept	−0.54 ± 0.25	−2.14	−1.08	−0.02	0.04
Population size^a^	−0.94 ± 0.08	−11.29	−1.11	−0.75	<0.001

^a^ Log‐transformed.

#### 3.3.2 Comparing the observed to theoretical relationships between population size and social structure

**FIGURE 2:**
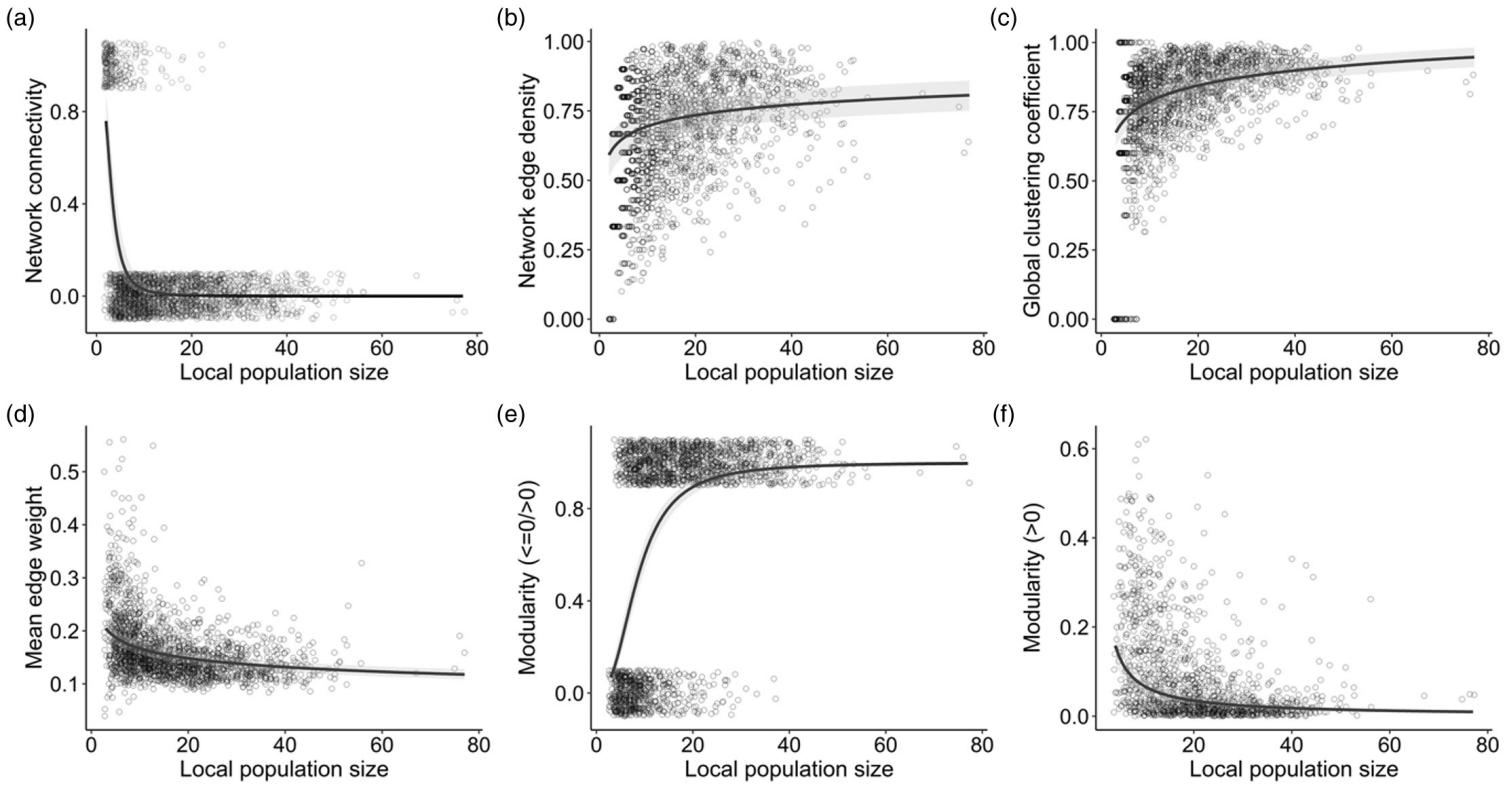
Predicted relationship between local population size and the observed social network structure. Shown are the predicted effects for (a) network connectivity (fully connected versus non‐fully connected networks), (b) network edge density, (c) global clustering coefficient, (d) average non‐zero edge weight, (e) modularity (contrasting values <=0 vs. >0) and (f) modularity values >0. Raw data of all weekly, local network metrics are shown as black dots, the predicted effect of the (G)LMMs is shown by the black line and the 95% CI as the grey ribbon. Predicted effects shown in all figures have been extracted using the package ‘effects’ (Fox et al., 2016) and have been back‐transformed. In panels (a and e), raw data have been jittered for visualization purposes.

**FIGURE 3:**
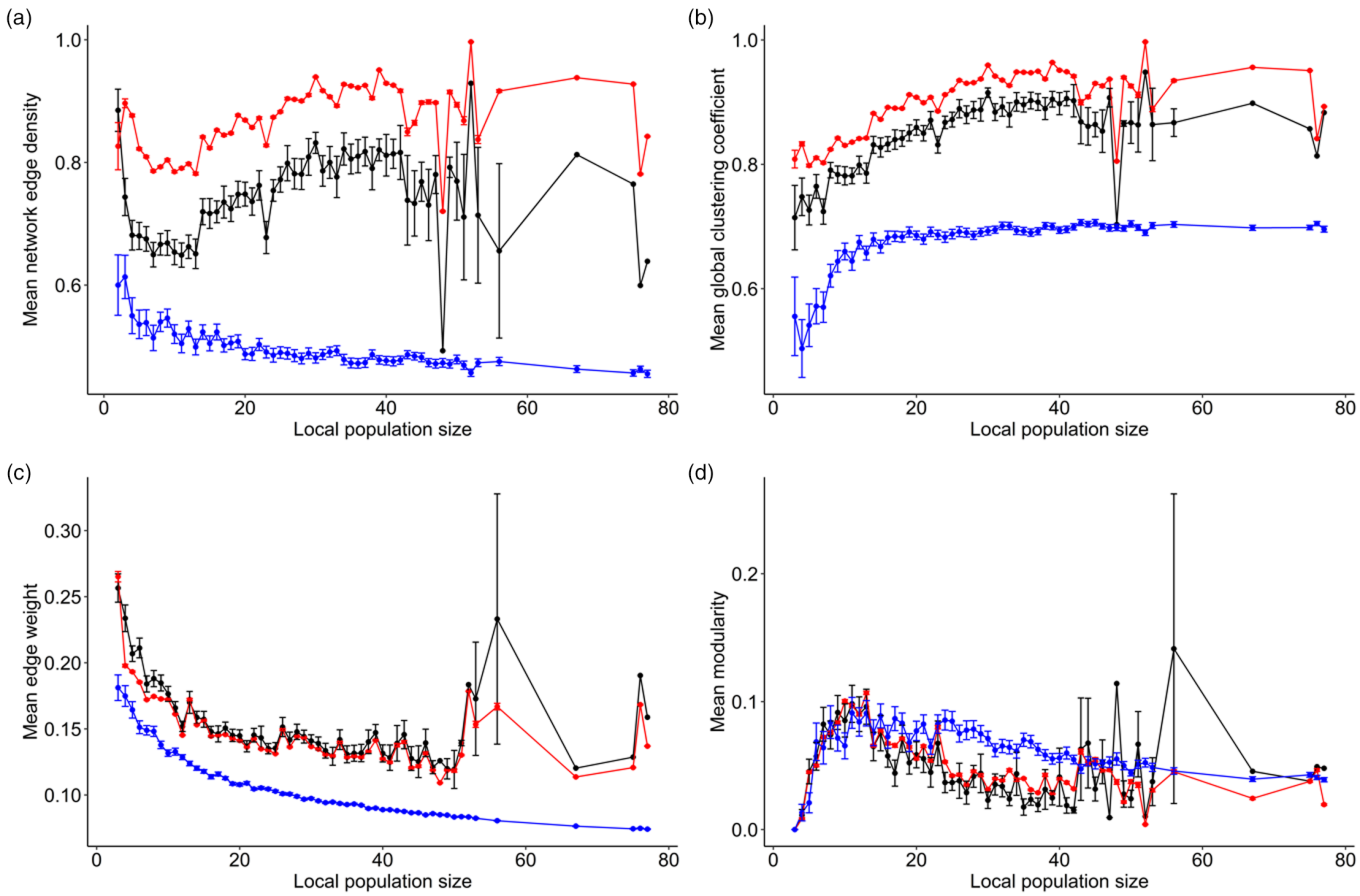
Comparison between the observed and theoretical relationship between global network metrics and local population size. Shown are the averages and standard errors of (a) network edge density, (b) global clustering coefficient, (c) mean edge weight and (d) modularity, and how these are expected to change with local population size. Each panel shows the expectation for three different models: a simple model (blue, no flocking or social preferences), a flocking‐only model (red, no social preferences) and the observed data (black).

When comparing the observed relationship between population size and network edge density (*N* = 2232) to the two models ignoring certain social features, we find that the ‘simple’ model produced on average the smallest values for network edge density (blue line in Figure 3a) while the ‘social foraging’ model produced the largest values (red line in Figure 3a). The observed data are in between the ‘simple’ and ‘social foraging’ model (black line in Figure 3a). For the observed data and the ‘social foraging’ model, network edge density was comparably high for very small population sizes (i.e. <10 individuals) which is caused by the increased occurrence of fully connected networks at smaller population sizes (note that fully connected networks have not been removed here for the comparison between observed and theoretical network structure). For the ‘simple’ model, network edge density decreased with increasing population size (Figure 3a). For both the ‘social foraging’ model and the observed data, network edge density initially decreased up to around 10 individuals (see also model results on observed network connectivity, Table 1, Figure 2a), followed by a slight increase up to about 30 individuals (Figure 3a, Table 1).

For all following comparisons, we removed networks with fewer than two connections (edges). The patterns for global clustering coefficient were overall very similar to the patterns observed for network edge density. The ‘simple’ model exhibited on average the smallest clustering coefficients and increased with increasing local population size up to approximately 10 individuals (blue line in Figure 3b). For the ‘social foraging’ model and the observed data, clustering coefficients on average slightly increased up until approximately 30 individuals (red and black line in Figure 3b, Table 1 for model results on observed data). Similarly, as for network edge density, the ‘social foraging’ model produced on average networks with larger global clustering coefficients than the observed data (Figure 3b).

All three models show a similar relationship between mean edge weight and population size, that is a decrease with increasing population size (Figure 3c). Both the observed data and the ‘social foraging’ model revealed an almost identical relationship between population size and mean edge weight, and the ‘simple’ model produced on average smaller mean edge weights (Figure 3c).

Social networks generated across all three models showed a similar relationship between modularity and population size, that is an initial increase in modularity up to approximately 10 individuals, followed by a decrease up to approximately 30/40 individuals (Figures 2e,f and 3d, Table 1). The ‘simple’ model produced similar values as the ‘social foraging’ model and the observed data (Figure 3d). Particularly for population sizes up to 10 individuals, all three models showed an almost identical relationship (Figure 3d). However, for population sizes larger than approximately 10 individuals, modularity dropped faster under the ‘social foraging’ model and the observed data (Figure 3d). For the ‘social foraging’ model and the observed data, modularity decreased to approximately 0.02 for population sizes of about 30/40 individuals (Figure 3d), whereas modularity remained at approximately 0.06 under the ‘simple’ model (blue line in Figure 3d). For both the ‘social foraging’ model and the observed data, the relationship between modularity and population size was almost identical (Figure 3d).

## 4 DISCUSSION

Here we show that local population size in great tits is highly consistent within distinct locations across weeks and years. Differences in population size were partly explained by the distance of the feeder location to the forest edge with locations closer to the edge recording on average more birds. In contrast to our prediction, we found no evidence that shrub‐cover density explained variation in local population size. Nevertheless, variation in population size affected the observed local social network structure; when comparing the relationship between social network structure and population size of the observed data to two datasets generated with models ignoring certain social processes, we reveal that social features such as the birds' preferences to forage in flocks and social preferences between birds (i.e. social relationships) mediated effects of population size on social network structure. This was especially the case for network properties concerning the density, clustering and strength of connections, while the extent of network fragmentation (i.e. modularity) was primarily driven by variation in local population size alone.

Variation in local population size affected local great tit social structure. Full network connectivity was much more likely at locations with smaller population sizes (with fewer than 10 individuals, Table 1, Figure 2a). This is presumably because great tits forage in flocks and cases of low population size may likely represent one loosely connected flock where all individuals are connected to each other at least once during that time‐frame. After excluding fully connected networks, both network edge density and global clustering coefficient increased with increasing population size (Table 1, Figure 2b,c) suggesting that the encounter probability between individuals at the feeder increased. In great tits, flock size increases with increasing local population size but saturates at population sizes of about 50/60 individuals (Farine et al., 2015). Larger flocks visiting a feeder may facilitate opportunities for social encounters if larger flocks have longer and more foraging bouts. Furthermore, locations with larger population sizes may represent a ‘preferred’ foraging patch which may increase the frequency with which individuals visit a feeder, leading to more opportunities for social encounters and thus increased network edge densities. The mean edge weight decreased with increasing population size (Table 1, Figure 2d). This is because with increasing population size, the number of social connections increases and thus individuals will have on average more but weaker connections to conspecifics resulting in lower edge weights. Modularity was overall very small but highest at locations of around 10 individuals (Figures 2e,f and 3d). Population sizes of about 10 individuals may represent few distinct flocks that are less likely to encounter each other at the feeder resulting in higher modularity. Cases with fewer than 10 individuals rather represent single flocks resulting in no network fragmentation. Modularity decreased with population sizes larger than 10 individuals and remained relatively constant around population sizes of 40/50 individuals and larger (Figure 2f). Note that we do have very little data on population sizes larger than 50 individuals. Thus, results for very large population sizes should be interpreted with caution. The decrease in network fragmentation with increasing population size may result from an increase in flock size, foraging duration and potentially visiting frequencies at the feeder, which will require further investigation in future studies.

Comparing the observed relationship between local population size and social structure to two models removing social features revealed that under the ‘simple’ model, network edge density decreased with increasing population size, while for the observed data and the ‘social foraging’ model, edge density first decreased but then increased again (Figure 3a). Furthermore, the ‘simple’ model produced on average lower values compared to the observed data and the ‘social foraging’ model (Figure 3a). This suggests that primarily social features, such as the preference for social foraging, drive the extent of realized connections. Interestingly, the ‘social foraging’ model produced on average very similar but slightly larger network edge densities compared to the observed data (Figure 3a) which suggests that also social preferences between birds (e.g. between breeding partners, Firth et al., 2015; Psorakis et al., 2012) shaped the extent of realized social connections. Hence, even in the very open and long‐thought unstructured society of great tits, individuals seem to continue to express their social preferences across population sizes, and maybe even are increasingly resistive to ‘random’ connections. The global clustering coefficient increased with population size in all three models but the ‘simple’ model produced on average the lowest values compared to the other two models (Figure 3b). This suggests again that social features may drive the observed clustering. Similar to the results on network edge density, the ‘social foraging’ model produced on average similar but larger values compared to the observed data suggesting that social preferences between birds drive the extent of clustering (Figure 3b). For the mean edge weight, all models showed a similar relationship, that is a decrease in mean edge weight with increasing population size (Figure 3c). This is because with increasing population size, the number of social connections an individual has increases and thus individuals will have on average more but weaker connections to others. Across different population sizes, the ‘simple’ model produced on average networks with smaller mean edge weights compared to the ‘social foraging’ model and the observed data (Figure 3c). Comparing the observed data to the ‘social foraging’ model did not reveal a difference (Figure 3c). Finally, all three models showed a similar relationship between modularity and population size, that is an initial increase up to approximately 10 individuals followed by a decrease (Figure 3d). In contrast to the other three network metrics, the ‘simple’ model resulted in similarly fragmented networks as for the observed data and the ‘social foraging’ model (Figure 3d), suggesting that social features such as with whom to forage with may only play a minor role in mediating network modularity. Taken together, our findings indicate that social features such as social foraging and social preferences between birds strongly impact network properties that relate to the extent and strength of social connections across different population sizes, but may play a rather minor role in shaping network modularity.

To what extent demographic features such as population size and density impact the observed social structure will ultimately depend on a species' social system. Great tits are highly gregarious species foraging in fission–fusion societies where individuals frequently leave and join groups (Ekman, 1989). However, many species are more solitary, mostly foraging alone (such as sleepy lizards, Leu et al., 2016 and desert tortoise, *Gopherus agassizii*, Sah et al., 2016) or exhibit much more territorial and stable social groups with little fission and fusion such as many primate and carnivore species (Holekamp et al., 2007; Kappeler & van Schaik, 2002). We find that network edge density increased with increasing population size (Figure 3a) which is in contrast to findings in other species such as in yellow‐bellied marmots (Maldonado‐Chaparro et al., 2015) or many primate groups (Sueur et al., 2011) where network edge density decreases with increasing group size. Great tits freely mix with conspecifics and may prefer to forage at locations with high densities, thus increasing their visiting frequency and the probability of social encounters. In fact, group sizes have been reported to increase with increasing local population size in great tits but saturated around population sizes of 50/60 individuals (Farine et al., 2015). Together with higher rates of feeder visits this may result in overall increased network edge densities. Species in more stable groups may be more territorial and actively avoid others in which case increases in population size may not necessarily increase group sizes and social encounter rates to such an extent that it increases overall network edge density. Similarly, in solitary species we may expect to see a similar pattern as observed in the ‘simple’ model (Figure 3a). While increases in population size may generally increase the probability of social encounter between individuals, increases in population size may impact the number of realized connections to a smaller extent than it impacts the number of possible connections and thus leads to overall decreased network edge densities. Differences in the relationship between network edge density and population size may also be due to methodological differences depending on the context and definition of social connections. For example, our social networks were generated from the birds' foraging associations, while other studies examined social interactions (such as body contact and grooming) to create social networks. For future work, it would be interesting to explore how variation in population size shapes social structure across species with different social systems and how such effects may be mediated by social features. Finally, great tits forage with other species (in our population, they mostly forage with blue tits [*Cyanistes caeruleus*], marsh tits [*Poecile palustris*], coal tits [*Periparus ater*] and nuthatches [*Sitta europaea*]) that also contribute to the social configuration of a foraging flock (Farine et al., 2015). Here, it would be interesting to examine how variation in the population size of these species influences their foraging associations and subsequent mixed‐species social networks. For example, increased population sizes may increase competition leading to more flocks with higher proportions of conspecifics and subsequently more assorted social networks.

Our study also highlights potential methodological consequences for how we measure and interpret social structure. For instance, research on small birds often utilizes feeders to infer foraging associations between individuals (Brandl et al., 2019; Dunning et al., 2023; Evans & Morand‐Ferron, 2019; Heinen et al., 2021; Jones et al., 2019; Psorakis et al., 2012). In these studies, the spatiotemporal availability of feeders often varies. For example, studies differ in the food and number of feeders provided, the distance between them and the time over which food is available. Resource abundance and distribution fundamentally impact the spatiotemporal distribution of individuals and subsequently the rate of social encounters. The specific setup chosen for a study may thus likely have consequence on the inferred social structures (Evans & Morand‐Ferron, 2019). For instance, comparing a setup with five feeders to a setup with only one feeder, providing the same space and equal population size, will likely lead to higher direct social encounter rates at the setup with the single feeder (Ferreira et al., 2020). In addition, methods to infer social associations may be sensitive to variation in population size and density. For instance, here we use Gaussian mixture models (Psorakis et al., 2012, 2015) to infer non‐overlapping grouping events (i.e. flocking events). Very high population sizes and activity at feeders may hinder the ability to correctly infer such grouping events and may thus overestimate group size and the co‐occurrence between individuals. Hence, the setup and methods to infer social associations should be carefully chosen considering a species' biology (e.g. degree of gregariousness), and social structures should always be interpreted with the data collection method in mind.

An increasing number of studies demonstrate how habitat features can shape social structure. For instance, in Australian sleepy lizards (*Tiliqua rugosa*) increased habitat structural complexity led to, on average, denser networks (Leu et al., 2016) and in red‐backed fairywrens (*Malurus melanocephalus*) habitat changes due to wildfires affected the social connectivity between birds (Lantz & Karubian, 2017). Habitat‐dependent effects on social structure may be mediated by changes in population size and density. For instance, spatiotemporal variation in the habitat led to local variation in population density influencing individual social connectivity in wild red deer (*Cervus elaphus*; Albery et al., 2021) and elk (*Cervus canadensis*; Webber & Vander Wal, 2020) where individuals at higher densities had on average more social connections. Our study supplements these findings by demonstrating how habitat features such as the distance to the forest edge influence variation in local population size and subsequently features of the global social network structure. What exactly drives these edge effects in our study will need further investigation. It is likely that the edge effect is related to vegetation features because understorey density is often higher at forest edges (Euskirchen et al., 2001; Harper & Macdonald, 2001; Šálek et al., 2010) and thus may attract more birds. Furthermore, immigration might be higher at forest edges (Wilkin et al., 2006) potentially leading to higher bird densities. Contrary to our predictions, shrub‐cover density did not predict differences in local population size at feeders (Table S1, Figure S4). However, the shrub‐cover data provided by Kirby et al. (2014) have not been collected for the purpose of our study and thus may not accurately reflect the vegetation structure around the feeder locations (i.e. feeder locations overlapped with the vegetation sampling plots to different extents, Figure S1). Therefore, research with more detailed measures on vegetation features at the distinct feeder locations may provide different results.

Density‐dependent variation in local social structure can have implications for a range of population processes such as facilitating or hindering the local emergence and spread of novel behaviours (Somveille et al., 2018) or diseases (Hu et al., 2013), and can impact the expression and evolution of social traits (Montiglio et al., 2018). Therefore, it is important to generate a better understanding of the interplay between the physical and social environment, and the exact mechanisms shaping variation in social structure (e.g. social decisions vs. habitat features). This is also of particular importance given that humans increasingly modify the environment animals live in. From urbanization and habitat fragmentation, to hunting activities, and changes in resource availability (e.g. agriculture and food subsidies such as bird feeders), humans alter habitat features in spurious ways which can have consequence for animal behaviour (Wilson et al., 2020) and social structure (Blumstein et al., 2022). For example, human‐induced alterations in the physical environment can fundamentally impact when and where animals move (Tucker et al., 2018) shaping the spatiotemporal distribution of animals and local population properties. In grizzly bears (*Ursus arctos*), local population density was considerably lower in areas with high road densities (Lamb et al., 2018), food subsidies such as landfills often attract large numbers of individuals leading to high local densities (Oro et al., 2013), and in our study local great tit population sizes were higher at forest edges (a result of human‐induced habitat fragmentation due to agriculture). Given the importance of the social environment in shaping individual survival and several ecological and evolutionary dynamics, a better understanding of how (human‐induced) environmental changes affect animal social structure is crucial.

## AUTHOR CONTRIBUTIONS

Kristina B. Beck was involved in conceptualization, data analysis, visualization and writing—original draft. Damien R. Farine, Josh A. Firth and Ben C. Sheldon were involved in conceptualization and writing—review and editing.

## CONFLICT OF INTEREST STATEMENT

The authors declare no competing interests.

## STATEMENT ON INCLUSION

Our study brings together authors from different countries, including scientists based in the country where the study was carried out. All authors were engaged early on with the research and study design to ensure that the diverse sets of perspectives they represent were considered from the onset.

## Supporting information


**Figure S1.** Map of the study site Wytham Woods. Black dots show all 65 feeder locations, red asterisk show the locations of the 164 10 m × 10 m quadrats at which shrub‐cover density data have been collected in 2012. Feeders are approx. 250 m apart from each other and locations remained consistent across years.
**Figure S2.** Histogram showing the frequency of the values for network edge density (i.e. the ratio between realized and possible connections) across all local, weekly social networks. The data distribution reveals a large peak for fully connected networks (i.e. network edge density of 1).
**Figure S3.** Histogram showing the frequency of the values for modularity (i.e. the extent of network fragmentation) across all local, weekly social networks. The data distribution reveals a large peak for modularity values around 0. Therefore, we first aimed at modelling all those values contributing to the increased left bar, followed by a model for all remaining values. Simply selecting values <=0 did not change the distribution substantially. Therefore, we selected the value 0.0001 as a different threshold.
**Figure S4.** Predicted effects between local population size and shrub‐layer density (left) and distance to forest edge (right). Raw data are shown as black dots, predicted relationship is shown by the black line and the grey‐shaded ribbon shows the 95% Confidence Interval.
**Table S1.** Results of the LMM examining the effect of shrub‐layer density and distance to the forest edge on local population size. Shown are estimates ± standard errors (SE), the test statistic z, 2.5% and 97.5% Confidence Intervals (CI) and the p value (*p*). Location and week nested within year were set as random effects (Variance and Standard deviation: Location = 0.30, 0.55; Week:Year = 0.01, 0.10).
**Table S2.** Results of the LMM examining the effect of shrub‐layer density and distance to the forest edge on local population size when considering a 100 m radius around the feeder to infer shrub‐layer density instead of 30 m. Shown are estimates ± standard errors (SE), the test statistic z, 2.5% and 97.5% Confidence Intervals (CI) and the p value (*p*). Location and week nested within year were set as random effects (Variance and Standard deviation: Location = 0.30, 0.54; Week:Year = 0.01, 0.10).
**Table S3.** Correlation coefficients between the four global network metrics (after excluding fully connected networks and cases where not at least two social connections existed, N_Networks_ = 1939).
**Table S4.** Results of the (G)LMMs examining the effect of local population size on the observed social network structure (network connectivity, square‐transformed network edge density, square‐transformed global clustering coefficient, log‐transformed mean edge weight, modularity (binomial, (<=0/>0) and log‐transformed modularity (for values >0). Shown are estimates ± standard errors (SE), the test statistic (i.e. *z* and *t* statistics), 2.5% and 97.5% Confidence Intervals (CI) and the p value (*p*).

## Data Availability

Data and code available from the Open Science Framework https://doi.org/10.17605/OSF.IO/PNJFW (Beck et al., 2023).
