# Supplementary material for: Variation in local population size predicts social network structure in wild songbirds
Source: J Anim Ecol. 2023 Oct 13;92(12):2348–62. doi: 10.1111/1365-2656.14015 (PMC10952437; doi:10.1111/1365-2656.14015)
Supplement: Supplementary file 1 — Figure S1. Map of the study site Wytham Woods. Black dots show all 65 feeder locations, red asterisk show the locations of the 164 10 m × 10 m quadrats at which shrub‐cover density data have been collected in 2012. Feeders are approx. 250 m apart from each other and locations remained consistent across years. Figure S2. Histogram showing the frequency of the values for network edge density (i.e. the ratio between realized and possible connections) across all local, weekly social networks. The data distribution reveals a large peak for fully connected networks (i.e. network edge density of 1). Figure S3. Histogram showing the frequency of the values for modularity (i.e. the extent of network fragmentation) across all local, weekly social networks. The data distribution reveals a large peak for modularity values around 0. Therefore, we first aimed at modelling all those values contributing to the increased left bar, followed by a model for all remaining values. Simply selecting values <=0 did not change the distribution substantially. Therefore, we selected the value 0.0001 as a different threshold. Figure S4. Predicted effects between local population size and shrub‐layer density (left) and distance to forest edge (right). Raw data are shown as black dots, predicted relationship is shown by the black line and the grey‐shaded ribbon shows the 95% Confidence Interval. Table S1. Results of the LMM examining the effect of shrub‐layer density and distance to the forest edge on local population size. Shown are estimates ± standard errors (SE), the test statistic z, 2.5% and 97.5% Confidence Intervals (CI) and the p value (p). Location and week nested within year were set as random effects (Variance and Standard deviation: Location = 0.30, 0.55; Week:Year = 0.01, 0.10). Table S2. Results of the LMM examining the effect of shrub‐layer density and distance to the forest edge on local population size when considering a 100 m radius around the feeder to infer shrub‐layer densit [file JANE-92-2348-s001.docx]

**Supporting information**


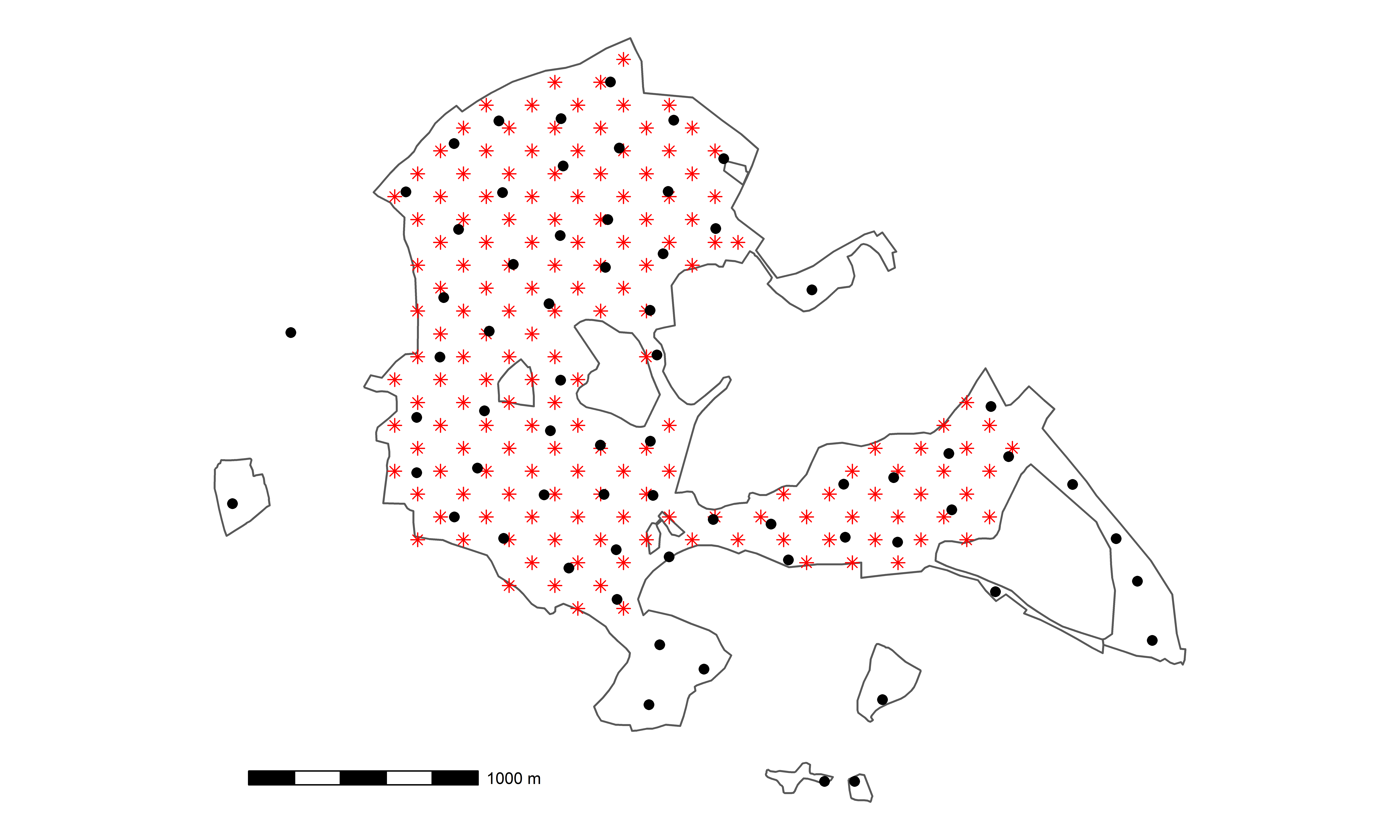


**Figure S1.** Map of the study site Wytham Woods. Black dots show all 65 feeder locations, red asterisk show the locations of the 164 10m x 10m quadrats at which shrub-cover density data have been collected in 2012. Feeders are approx. 250m apart from each other and locations remained consistent across years.


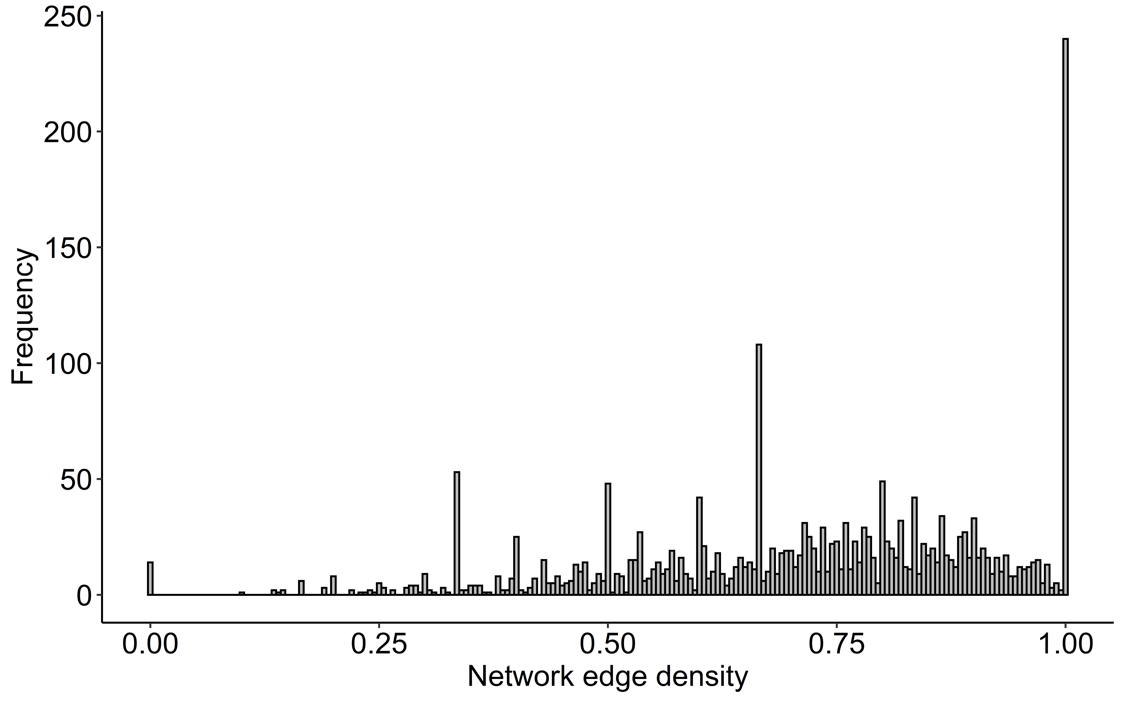


**Figure S2.** Histogram showing the frequency of the values for network edge density (i.e. the ratio between realized and possible connections) across all local, weekly social networks. The data distribution reveals a large peak for fully connected networks (i.e. network edge density of 1).


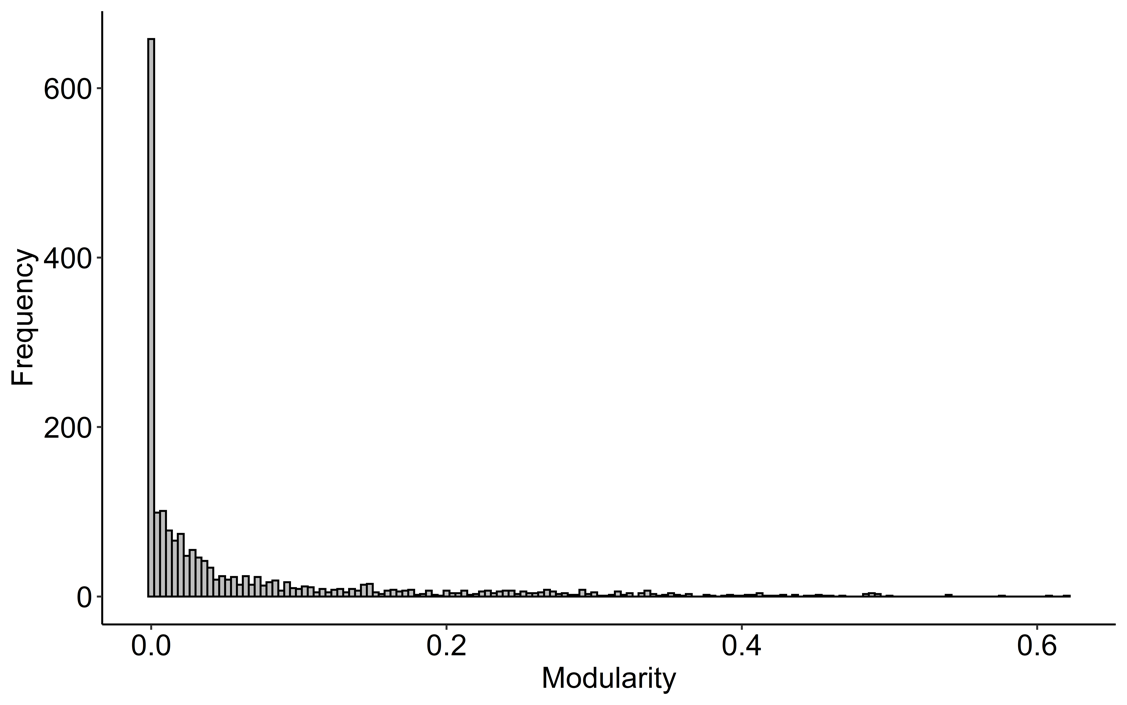


**Figure S3.** Histogram showing the frequency of the values for modularity (i.e. the extent of network fragmentation) across all local, weekly social networks. The data distribution reveals a large peak for modularity values around 0. Therefore, we first aimed at modelling all those values contributing to the increased left bar, followed by a model for all remaining values. Simply selecting values <=0 did not change the distribution substantially. Therefore, we selected the value 0.0001 as a different threshold.


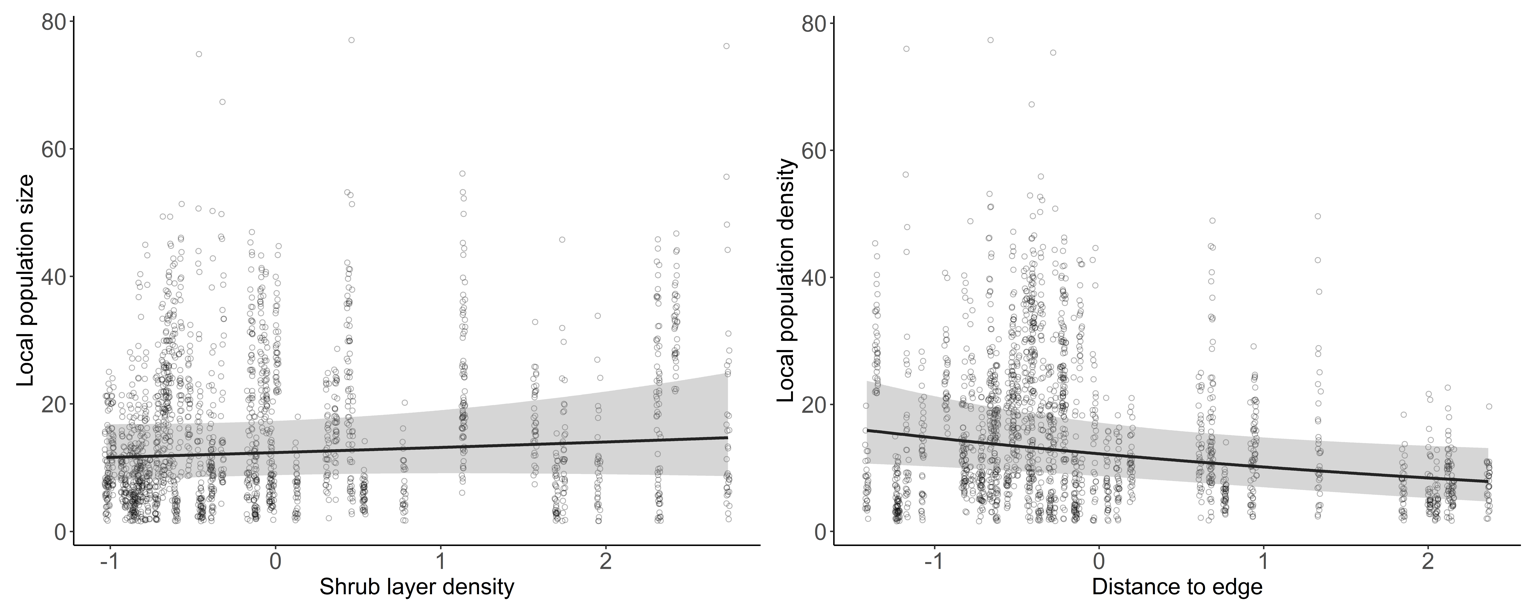


**Figure S4**. Predicted effects between local population size and shrub-layer density (left) and distance to forest edge (right). Raw data are shown as black dots, predicted relationship is shown by the black line and the grey-shaded ribbon shows the 95% Confidence Interval.

**Table S1**. Results of the LMM examining the effect of shrub-layer density and distance to the forest edge on local population size. Shown are estimates ± standard errors (SE), the test statistic z, 2.5% and 97.5% Confidence Intervals (CI) and the p value (P). Location and week nested within year were set as random effects (Variance and Standard deviation: Location=0.30, 0.55; Week:Year=0.01, 0.10).

|  | **Estimate ± SE** | **z** | **2.5% CI** | **97.5% CI** | **P** |
| --- | --- | --- | --- | --- | --- |
| Intercept | 2.50 ± 0.17 | 14.39 | 2.16 | 2.84 | <0.001 |
| Shrub-layer density* | 0.06 ± 0.08 | 0.83 | -0.09 | 0.22 | 0.41 |
| Distance to edge* | -0.19 ± 0.08 | -2.28 | -0.35 | -0.03 | 0.02 |

* standardized by subtracting the mean and dividing the standard deviation.

**Table S2**. Results of the LMM examining the effect of shrub-layer density and distance to the forest edge on local population size when considering a 100m radius around the feeder to infer shrub-layer density instead of 30m. Shown are estimates ± standard errors (SE), the test statistic z, 2.5% and 97.5% Confidence Intervals (CI) and the p value (P). Location and week nested within year were set as random effects (Variance and Standard deviation: Location=0.30, 0.54; Week:Year=0.01, 0.10) .

|  | **Estimate ± SE** | **z** | **2.5% CI** | **97.5% CI** | **P** |
| --- | --- | --- | --- | --- | --- |
| Intercept | 2.50 ± 0.17 | 14.41 | 2.16 | 2.84 | <0.001 |
| Shrub-layer density* | 0.09 ± 0.08 | 1.14 | -0.06 | 0.24 | 0.26 |
| Distance to edge* | -0.18 ± 0.08 | -2.20 | -0.34 | -0.02 | 0.03 |

* standardized by subtracting the mean and dividing the standard deviation.

**Table S3**. Correlation coefficients between the four global network metrics (after excluding fully connected networks and cases where not at least two social connections existed, N_Networks_=1939).

|  | **Average edge weight** | **Global clustering coefficient** | **Network edge density** |
| --- | --- | --- | --- |
| **Global clustering coefficient** | -0.14 |  |  |
| **Network edge density** | -0.21 | 0.67 |  |
| **Modularity** | 0.31 | -0.34 | -0.56 |

**Table S4.** Results of the (G)LMMs examining the effect of local population size on the observed social network structure (network connectivity, square-transformed network edge density, square-transformed global clustering coefficient, log-transformed mean edge weight, modularity (binomial, (<=0/>0) and log-transformed modularity (for values >0). Shown are estimates ± standard errors (SE), the test statistic (i.e. z and t statistics), 2.5% and 97.5% Confidence Intervals (CI) and the p value (P).

|  | **Estimate ± SE** | **Test statistic** | **2.5% CI** | **97.5% CI** | **P** |
| --- | --- | --- | --- | --- | --- |
| **Network connectivity**  (N_Networks_=1037) |  |  |  |  |  |
| Intercept | 2.44 ± 0.53 | 4.57 | 1.39 | 3.49 | <0.001 |
| Population size* | -2.46 ± 0.22 | -11.40 | -2.88 | -2.03 | <0.001 |
|  |  |  |  |  |  |
| **Network edge density**  (N_Networks_=954) |  |  |  |  |  |
| Intercept | 0.20 ± 0.06 | 3.08 | 0.06 | 0.33 | 0.003 |
| Population size* | 0.12 ± 0.01 | 9.40 | 0.09 | 0.15 | <0.001 |
|  |  |  |  |  |  |
| **Clustering coefficient**  (N_Networks_=919) |  |  |  |  |  |
| Intercept | 0.27 ± 0.05 | 5.46 | 0.17 | 0.37 | <0.001 |
| Population size* | 0.15 ± 0.01 | 12.55 | 0.12 | 0.17 | <0.001 |
|  |  |  |  |  |  |
| **Mean edge weight**  (N_Networks_=919) |  |  |  |  |  |
| Intercept | -1.29 ± 0.06 | -21.87 | -1.41 | -1.18 | <0.001 |
| Population size* | -0.21 ± 0.02 | -11.54 | -0.24 | -0.17 | <0.001 |
| **Modularity (<=0/>0)**  (N_Networks_=919) |  |  |  |  |  |
| Intercept | -4.80 ± 0.52 | -9.21 | -5.82 | -3.78 | <0.001 |
| Population size* | 2.28 ± 0.20 | 11.24 | 1.88 | 2.68 | <0.001 |
|  |  |  |  |  |  |
| **Modularity (>0)**  (N_Networks_=655) |  |  |  |  |  |
| Intercept | -0.09 ± 0.37 | -0.24 | -0.84 | 0.67 | 0.81 |
| Population size* | -1.11 ± 0.11 | -10.51 | -1.34 | -0.87 | <0.001 |
|  |  |  |  |  |  |

* Log-transformed
